# Auditory processing in children and adolescents in situations of risk and vulnerability

**DOI:** 10.1590/S1516-31802012000300004

**Published:** 2012-07-12

**Authors:** Cristina Ferraz Borges Murphy, Fernanda Pontes, Luciene Stivanin, Erica Picoli, Eliane Schochat

**Affiliations:** I MD, PhD. Speech Therapist and Postdoctoral Researcher in the field of auditory processing at Universidade de São Paulo (USP), São Paulo, Brazil.; II MD. Speech Therapist, Department of Physical Therapy, Speech Therapy and Occupational Therapy, Universidade Estadual Paulista Julio de Mesquita Filho (Unesp), Botucatu, São Paulo, Brazil.; III MD. Speech Therapist and Postgraduate Student in Rehabilitation Science, Universidade de São Paulo (USP), São Paulo, Brazil.; IV MD, PhD. Associate Professor, Department of Physical Therapy, Speech Therapy and Occupational Therapy, Faculdade de Medicina da Universidade de São Paulo (FMUSP), São Paulo, Brazil.

**Keywords:** Homeless youth, Social problems, Evoked potentials, auditory, brain stem, Auditory perceptual disorders, Cognition, Menores de rua, Problemas sociais, Potenciais evocados auditivos do tronco encefálico, Transtornos da percepção auditiva, Cognição

## Abstract

**CONTEXT AND OBJECTIVE:**

Children and adolescents who live in situations of social vulnerability present a series of health problems. Nonetheless, affirmations that sensory and cognitive abnormalities are present are a matter of controversy. The aim of this study was to investigate aspects to auditory processing, through applying the brainstem auditory evoked potential (BAEP) and behavioral auditory processing tests to children living on the streets, and comparison with a control group.

**DESIGN AND SETTING:**

Cross-sectional study in the Laboratory of Auditory Processing, School of Medicine, Universidade de São Paulo.

**METHODS:**

The auditory processing tests were applied to a group of 27 individuals, subdivided into 11 children (7 to 10 years old) and 16 adolescents (11 to 16 years old), of both sexes, in situations of social vulnerability, compared with an age-matched control group of 10 children and 11 adolescents without complaints. The BAEP test was also applied to investigate the integrity of the auditory pathway.

**RESULTS:**

For both children and adolescents, there were significant differences between the study and control groups in most of the tests applied, with significantly worse performance in the study group, except in the pediatric speech intelligibility test. Only one child had an abnormal result in the BAEP test.

**CONCLUSIONS:**

The results showed that the study group (children and adolescents) presented poor performance in the behavioral auditory processing tests, despite their unaltered auditory brainstem pathways, as shown by their normal results in the BAEP test.

## RESUMO

**CONTEXTO E OBJETIVO:**

Crianças e adolescentes que vivem em situação de vulnerabilidade social apresentam uma série de problemas de saúde. Apesar disso, ainda é controversa a afirmação sobre a existência de alterações cognitivas e/ou sensoriais. O objetivo deste estudo foi investigar aspectos relacionados ao processamento auditivo, através da aplicação de testes de potencial evocado auditivo de tronco encefálico (PEATE) e avaliação comportamental do processamento auditivo em crianças em situação de rua, comparando a um grupo controle.

**TIPO DE ESTUDO E LOCAL:**

Estudo transversal no Laboratório de Processamento Auditivo, Faculdade de Medicina da Universidade de São Paulo.

**MÉTODOS:**

Os testes de processamento auditivo foram aplicados em um grupo de 27 indivíduos, subdivididos em grupos de 11 crianças (7 a 10 anos) e 16 adolescentes (11 a 16 anos) de ambos os sexos, em situação de vulnerabilidade social, e comparado a um grupo controle, formado por 21 crianças, subdivididas em grupos de 10 crianças e 11 adolescentes, pareados por idade, sem queixas. Também se aplicou os PEATE para investigação da integridade da via auditiva.

**RESULTADOS:**

Para ambas as faixas etárias, foram encontradas diferenças significantes entre grupos estudo e controle para a maioria dos testes aplicados, sendo que o grupo estudo apresentou desempenho estatisticamente pior do que o controle para todos os testes, exceto para o teste *pediatric speech intelligibility*. Apenas uma criança apresentou resultado alterado para os PEATE.

**CONCLUSÕES:**

Os resultados demonstraram pior desempenho do grupo estudo (crianças e adolescentes) para os testes comportamentais de processamento auditivo, apesar de estes apresentarem integridade da via auditiva em nível de tronco encefálico, demonstrada pela normalidade nos resultados do PEATE.

## INTRODUCTION

Children and adolescents who grow up in socially vulnerable situations comprise a group with special characteristics. They spend at least part of the day in the streets, alone or in groups, have the appearance of being abandoned, with a visible lack of hygiene, and perform humble tasks with the aim of subsistence for themselves and their families.^1^ In Brazil, a United Nations report in 2009 emphasized the concern about remedying the problem of children living in the streets, where they become vulnerable to abuse, including sexual abuse and other forms of exploitation.^2^

A series of health problems, mainly of a physical nature, has been described in this population. According to Weinreb et al.,^3^ these children are four times more likely to have asthma, have twice as many ear infections and present nutritional problems^4^ and delayed growth.^5^ Language abnormalities have also been described,^6^ as have psychological disorders.^7^ Bassuk and Rubin,^7^ for example, reported high incidence of depression and anxiety. In addition to descriptions of physical and psychological disorders, a relationship between chronic physical problems and development alterations in these children has been shown.^8^^,^^9^ Cutuli et al.,^9^ for example, investigated the prevalence of asthma and the correlation with behavioral abnormalities. These authors found that cases of asthma were three times more prevalent than the national average, and that they showed a correlation with symptoms of attention deficit and hyperactivity.

With regard to cognitive factors, few studies have been published. Among the few that are available, the results are known to be controversial.^10^^,^^11^^,^^12^^,^^13^ Rubin et al.,^11^ for example, did not find any performance differences in non-verbal tests when comparing street children with children who have homes, despite finding great differences between the groups in terms of academic functions (reading and spelling, among other functions). In contrast, other studies^12^^,^^13^ found a performance difference in tests mainly involving vocabulary, between children with and without homes.

Another matter that is neglected in this population and that also, indirectly, involves cognitive processes concerns the manner in which sensory stimuli are interpreted. In the case of auditory stimuli, this involves processing of auditory information. According to Katz et al.,^14^ auditory processing is what people do with what they hear. Cognitive functions relating to attention and memory are also involved and need to be unimpaired for the auditory processing to be executed successfully.^15^ Tests known as “auditory processing tests”, for example, aim to investigate auditory functions through analyzing specific auditory skills such as localization, discrimination and ordering of different types of sound. Thus, in order for the performance in these tests to have results that can be considered to be “expected”, it is necessary not only for the auditory pathway to be complete, but also for the different cognitive functions to be complete.

Several studies^16^^,^^17^^,^^18^^,^^19^ have investigated possible risk factors for auditory processing disorders. The factors implicated have included histories of ear infections as well as immaturity of the auditory system due to environmental deprivation. Moreover, the consequences of such disorders, when not remedied, have also been investigated.^20^^,^^21^^,^^22^^,^^23^ One theory that has been studied to a moderate extent is that abnormalities in oral language and learning, like dyslexia, may arise from such disorders.^22^^,^^24^

There are no descriptions in the relevant literature about investigations of auditory processing in this population. Nevertheless, we are aware of its importance, given the problems that disorders of auditory processing can cause and the risk factors involved, which unfortunately are present among street children. This being so, the hypothesis that we put forward was that such children would present abnormalities alterations in tests that could be applied. The results from such tests could then be discussed afterwards taking into account the existing relevant literature. Furthermore, considering the fact that children and adolescents can experience different stressful life events, these two groups could be analyzed separately.

## OBJECTIVE

The aim of this study was to investigate aspects of auditory processing, through application of the brainstem auditory evoked potential (BAEP) and behavioral auditory processing tests, among at-risk children and adolescents in situations of social vulnerability, in comparison with a control group.

## METHOD

### Research design and participants

This cross-sectional study was conducted in the Laboratory of Speech and Language Investigation in Auditory Processing, within the Speech and Language Therapy Course of the School of Medicine, University of São Paulo (Faculdade de Medicina da Universidade de São Paulo, FMUSP). The study was approved by the Ethics Committee for Research Project Analysis (Comissão de Ética para Análise de Projetos de Pesquisa, CAPPesq) of the Clinical Directorate of Hospital das Clínicas, FMUSP, under research protocol 3849, project 0020/10.

The study group comprised 11 children (7 to 10 years old) and 16 adolescents (11 to 16 years old) who were in socially vulnerable situations. They were recruited through the Equilibrium Program (Table 1), which was developed by the Psychiatric Institute of FMUSP. The Equilibrium Program aims to provide multidisciplinary intervention within the process of social-family reintegration of children, through a team composed of psychiatrists, speech therapists, physiotherapists, social workers, psychologists and educational psychologists. This team is responsible for psychosocial characterization of the subjects, taking into consideration the international classification of diseases (ICD). For example, this includes the maltreatment syndrome, which particularly includes abandonment, physical ill-treatment, sexual and physical abuse, removal from home during childhood, education in an institution, emotional neglect, inadequate family support, family history of mental illness, etc. At the time of this study, all the participants had been taken into shelters and were taking part in the interventions and activities developed through this program. The group was composed of 27 individuals in total, of whom 11 were girls and 16 were boys, with an average age of 11 years and six months. All the individuals had the social diagnosis of Removal from Home in Childhood (Z61.1).

**Table 1.** Characteristics of the study and control groups

Characteristics	Study group		Control group	
	Children	Adolescents	Children	Adolescents
Total	11	16	10	11
Gender				
Female	6	5	5	6
Male	5	11	5	5
Age, mean ± SD (years)	8.9 ± 1.3	13.2 ± 1.6	9.8 ± 1.3	13.1 ± 1.7
Diagnoses	Z61.1	Z61.1	No complaints	No complaints
Audiological evaluation	No abnormalities	No abnormalities	No abnormalities	No abnormalities
Presence of school complaints	100% of group	100% of group	0% of group	0% of group

The control group was composed of 10 children (7 to 10 years old) and 11 adolescents (11 to 16 years old), matched according to age (Table 1). The clinical history questionnaire was completed for all children in an interview with the child and his or her parent(s) or care giver(s). The interviews did not raise any concerns regarding hearing or listening, cognitive, psychological, neurological or ophthalmological problems, delays in oral language acquisition or any musical knowledge, for any of the children.

Both groups came to the Audiology Sector of FMUSP between May and November 2008, and underwent an audiological evaluation composed of meatoscopy, imitanciometry and assessment of the vocal and tonal audiometric threshold, in order to investigate the presence of peripheral audiological abnormalities. The individuals who presented abnormalities in these examinations were excluded from the study and referred to an appropriate specialized professional for treatment. This criterion was adopted so that peripheral abnormalities did not interfere with the results from the central auditory examinations.

After the audiological evaluation, both groups (study and control) underwent behavioral auditory processing tests. In addition to these tests, the brainstem auditory evoked potential (BAEP) test was also applied to the study group, in order to investigate the integrity of the auditory brainstem pathways, and make comparisons with expected standard values. No comparison with a control group was made in this case, given that the expected standard values for this test are broadly accepted.^25^^,^^26^^,^^27^^,^^28^

### Examinations

#### Brainstem auditory evoked potentials (BAEPs)

The main function of this electrophysiological examination is to evaluate the integrity of the auditory pathway in the brainstem, in an objective manner.

To perform this examination, rarefaction polarity clicks were used, presented through TDH-39 earphones, monaurally at 80 dBnHL, at a presentation speed of 19.0 clicks per second, each of duration 0.1 milliseconds, using a total of 2000 stimuli. The absolute latencies of waves I, III and V and interpeaks I-III, III-V and I-V were checked and the normative values proposed for the click was taken.^25^

A portable Bio-logic (Traveler Express model) device was used, with electrodes coupled to the individual’s skin by means of electrolytic paste and held in place using adhesive tape. The electrodes were positioned on the forehead (Fz) and on the right and left mastoids (A2 and A1), in accordance with Jasper,^29^ with impedance values lower than 5 kohm.

#### Behavioral auditory processing tests

This evaluation was performed in accordance with the standards established by Pereira and Schochat.^30^ The auditory skills evaluated were:

- figure-background (pediatric speech intelligibility, PSI (Pediatric Speech Intelligibility), test): the skill of identifying the speech signal in the presence of other competing sounds;
- auditory closing (speech with noise test): recognizing the acoustic signal when parts of it are omitted;
- selective attention (non-verbal dichotic test): monitoring of a given significant auditory stimulus, even though primary attention is focused on another sensory stimulus or there is background noise present;
- binaural integration or synthesis (dichotic digit test): the skill of recognizing stimuli presented simultaneously or alternately in both ears;
- auditory memory (memory of nonverbal and verbal stimuli).

Presence of abnormalities in two or more auditory skills^31^ is considered to be the criterion for diagnosing auditory processing disorder. All the tests were performed in an acoustic booth, using a Grason-Stadler audiometer (model GSI-33) and a Sony compact disc player.

To analyze the data, the results from the behavioral auditory processing tests for the two groups were compared. The statistical analysis was done using the Mann-Whitney test and a significance level of 0.05 (5%). The BAEP results from the study group were compared with the expected standard values. With regard to the required sample size, the power analysis indicated that a sample size of 27 was needed in order to provide a power of 0.80 to detect a statistically significant difference in performance between the two groups (alpha level = 0.05).

## RESULTS

Table 2 shows the study group means, standard deviations and expected standards relating to the absolute latency values (ms) for waves I, III and V and interpeaks I-III, III-V and I-V of the BAEPs, for each ear. It can be seen that, considering the standard deviations,^25^ all the means obtained were within the expected standards. If each value obtained is taken individually for the 27 children considered, one of them presented abnormal results, with higher absolute latency and interpeak intervals preserved, which suggests that conductive hearing loss was present, despite the normal results from tonal audiometry and imitanciometry.

**Table 2.** Group means, standard deviations and expected standards^25^ relating to the absolute latency values (ms) of waves I, III and V and interpeaks I-III, III-V and I-V of brainstem auditory evoked potentials (BAEPs)

Latency	Mean ± standard deviation		Expected standard^25^
	Right ear	Left ear	
Wave I	1.55 ± 0.16	1.51 ± 0.16	1.54
Wave III	3.63 ± 0.17	3.64 ± 0.21	3.69
Wave V	5.57 ± 0.18	5.58 ± 0.19	5.54
Interpeaks I-III	2.08 ± 0.18	2.13 ± 0.19	2.14
Interpeaks III-V	1.94 ± 0.14	1.93 ± 0.15	1.86
Interpeaks I-V	4.02 ± 0.16	4.06 ± 0.14	4

Regarding the behavioral auditory processing tests, the performance comparisons between the two groups are shown in Table 3. The children and adolescents in the study group had significantly lower scores than shown by the control group, in all the tests except for the memory test for verbal sounds for the children and the PSI for both the children and the adolescents. There was a tendency towards significance for the dichotic digit test, for the adolescents.

**Table 3.** Comparison between study and control groups relating to the auditory processing tests

		Children			Adolescents		
		Study	Control	P	Study	Control	P
Auditory processing tests							
Memory test on verbal sounds (mean)		2.2 ± 1.0	2.7 ± 0.4	0.239	2.1 ± 1.0	2.9 ± 0.3	0.022
Memory test on nonverbal sounds (mean)		1.4 ± 1.2	2.8 ± 0.4	0.004	1.5 ± 0.9	3.0 ± 0	< 0.001
Pediatric speech intelligibility test							
Mean	RE	7.0 ± 2.0	8.5 ± 1.7	0.074	8.3 ± 1.7	8.1 ± 1.0	0.824
	LE	7.2 ± 1.2	7.5 ± 1.4	0.705	7.7 ± 1.5	7.6 ± 1.1	0.838
Speech with noise test							
Mean (%)	RE	61.6 ± 1.2	86.8 ± 1.4	< 0.001	63.2 ± 1.4	86.9 ± 0.9	< 0.001
	LE	66.0 ± 1.0	92.8 ± 0.7	< 0.001	66.2 ± 1.5	89.4 ± 0.8	< 0.001
Non-verbal dichotic test							
Mean	RE	8.0 ± 2.3	11.4 ± 0.5	< 0.001	9.2 ± 2.1	11.7 ± 0.4	0.001
	LE	9.1 ± 2.3	11.9 ± 0.3	0.002	9.9 ± 2.0	11.8 ± 0.4	0.007
Dichotic digit test							
Mean (%)	RE	74.7 ± 13.3	93.7 ± 2.4	< 0.001	79.5 ± 1.3	92.5 ± 3.5	0.006
	LE	70.4 ± 1.5	93.7 ± 3.5	< 0.001	75.3 ± 1.8	93.1 ± 6.3	0.006

RE = right ear; LE = left ear.

We also investigated the percentage distribution of the individuals in both groups in relation to the results from each behavioral auditory processing test, taking normalized values into consideration (Table 4). The PSI test and memory test for verbal sounds showed the lowest numbers of individuals with abnormalities, and the digit dichotic and non-verbal dichotic tests showed the largest numbers of individuals with abnormalities.

**Table 4.** Percentage distribution of children and adolescents with skill abnormalities in the study and control groups

Auditory processing tests	Study group		Control group	
	Percentage of children with skill abnormalities	Percentage of adolescents with skill abnormalities	Percentage of children with skill abnormalities	Percentage of adolescents with skill abnormalities
Memory test on nonverbal sounds	36.3%	43.7%	0%	0%
Memory test on verbal sounds	18.1%	18.7%	0%	0%
Pediatric speech intelligibility test	27.2%	6.2%	9%	0%
Non-verbal dichotic test	91.6%	62.5%	0%	0%
Dichotic digit test	90.9%	93.7%	18%	18.7%
Speech with noise test	81.8%	62.5%	0%	0%

**Figure 1.**
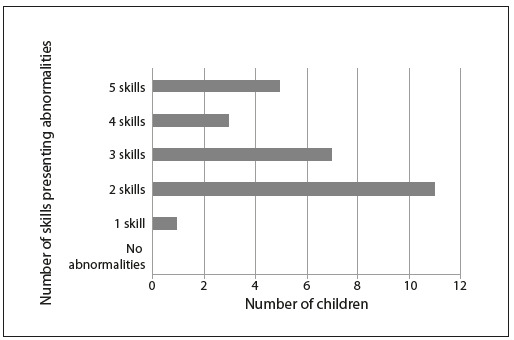
Distribution of the number of skills presenting abnormalities in the group.

Figure 1 shows the distribution of the number of skills for which abnormalities were presented in the study group, taking normalized values. All the children tested had an abnormality in at least one of the skills tested, with the majority having an abnormality in two skills. Considering the criterion used for the auditory processing diagnosis^31^ (abnormalities in two or more skills), it was found that among the 27 children evaluated, 26 (96.3%) had a diagnosis of auditory processing abnormality.

## DISCUSSION

The results showed that the study group presented significantly lower scores than shown by the control group, despite the unimpaired auditory brainstem pathways in the study group, as shown by the normality of the BAEP results. These results were the same for both age groups (children and adolescents).

According to several studies, both children and adolescents in socially vulnerable situations can experience different stressful life events. Whereas children have higher rates of asthma,^3^^,^^9^ for example, adolescents have complaints relating to accidents, learning^6^ and psychological problems^7^ like anxiety and depression. Nonetheless, the results from both tests, for both children and adolescents, were similar. There were differences relating to only one of the auditory processing tests (memory test for verbal sound), in which the adolescents showed significantly poor performance, compared with the control group. These results are perhaps related to the fact that both groups had deficits in the same characteristic, like cognition or attention, which are abilities related to performance in the auditory processing tests.

The unimpaired nature of the auditory brainstem pathway shows that, probably, none of the variables commonly correlated with the quality of life of this population, like malnutrition, ill-treatment and lack of stimulation were capable of interfering with these individuals’ neuroanatomical development. This result is odd, given that this pathway is not fully developed at birth and that, according to several studies, its appropriate development comes through stimulation, genetic background and general health.^32^^,^^33^^,^^34^^,^^35^

We did not know with any certainty for how long each child had been living in the streets and, therefore, the extent to which the study group had been insufficiently stimulated. Nevertheless, the results from the present study can be corroborated by studies performed on animals. In a study by Hartmann et al.,^36^ it was shown that the difference in tonotopic gradient of the auditory pathway between a group of adult congenitally deaf cats (i.e. with a complete lack of auditory experience) and a group of cats that could hear, was minimal. These researchers concluded that stimulation was not a preponderant factor in its development. Thus, we can assume that, even though the group in the present study had been in socially vulnerable situations since the first years of life, this factor cannot have been relevant to the development of the auditory pathway, especially regarding BAEPs, which become fully developed at two years of age.^25^^,^^26^^,^^27^^,^^28^^,^^32^

Different to the results from the BAEPs, most of the group presented abnormalities in the auditory processing tests (96.3%). Moreover, the disorder was present mainly in relation to dichotic tasks (digit dichotic and non-verbal dichotic tests) and the skill of auditory closing (speech with noise).

According to Keith and Anderson,^37^ dichotic listening tests are the best method for evaluating inter-hemispheric transfer of information and the maturity of the auditory nervous system. This is because, in this type of task, the contralateral pathways take priority in functioning. Thus, when a message is presented to the left ear, for example, it is transmitted to the opposite hemisphere (right), and subsequently crosses to the dominant hemisphere for language (left), through the corpus callosum. When the message is presented to the right ear, it is simply transmitted directly to the dominant hemisphere for language (left).

This difference in the route taken by the information leads to an advantage in the right ear, i.e. better performance for this ear in younger children or those with immaturity of the system. Moreover, the proportion of this advantage is indirectly related to this maturity, i.e. the greater the advantage is, the greater the indication of immaturity of the structures of the corpus callosum (the structure required for the inter-hemispheric transfer). In the present study, in the dichotic digit test, the study group presented lower-than-expected performance in the left year, thus showing a considerable advantage in the right ear: right ear 75.5% and left ear 72.6% for the children; and right ear 79.8% and left ear 74% for the adolescents. This result clearly shows that the adolescents in the study group presented immaturity of the system, in comparison with the control group. This immaturity can be correlated with episodes of recurring otitis, malnutrition and the very lack of stimulation. All these factors were probably present in this population, as already described in the introduction.^16^^,^^17^^,^^18^^,^^19^

Differing from BAEPs, in which the auditory pathway is analyzed neuroanatomically and physiologically, the pathway in the behavioral auditory processing tests is analyzed in a functional manner. Thus, the data in both the tests showed that in spite of the unimpaired nature of the auditory pathway, i.e. absence of anatomical and physiological structural abnormalities, the functioning of the pathway was abnormal. This result corroborates findings from other studies in which it was shown that exposure to stressful situations could impair cerebral functioning, thereby having an impact on the processing of the information. Among the cerebral areas that have been reported to be affected are the corpus callosum and areas in the left hemisphere^38^ and frontal areas,^39^ which are also areas related to processing of auditory information.

One of the hypotheses for this functional abnormality relates to immaturity, as already mentioned. According to Schochat and Musiek,^32^ even though the peripheral auditory system is practically ready at birth, myelination of the auditory pathways proceeds over the years, thus reflecting differences in BAEP values until reaching around two years of age and until 10-12 years of age in relation to auditory skills, as tested in the behavioral auditory processing tests. This observation may explain the different results from the two tests. It is possible that the situation to which the group was exposed at the time of this study may have influenced the maturation processes that were still occurring and which were shown in the behavioral auditory processing tests. On the other hand, maturation only needed to occur over the first two years of life for BAEPs to be completed, and it may have been that at that age, the study group was not yet exposed to all the risks to which these individuals were exposed at the time of this study.

In addition to the immaturity of the auditory system, another hypothesis can also be put forward in relation to the results from the auditory processing evaluation. As already mentioned in the introduction, for the performance results from the behavioral auditory processing evaluation to be at the “expected” level, not only has the auditory pathway to be unimpaired, but also several cognitive functions need to be unimpaired. The latter were not analyzed separately in the present study, but it is known that abnormalities of this type can occur in this group, although this finding is controversial.^10^^,^^11^^,^^12^^,^^13^ Some authors, for example, have mentioned a delay in development and weak performance in tests mainly involving vocabulary,^12^^,^^13^ while others have not found any abnormalities in relation to non-verbal skills.^11^

There is also controversy surrounding the skills considered in the formal tests.^1^ According to these authors, the cognitive development of these children should also be understood within their contextualized form, since their experiences in the streets allow different skills and forms of reasoning to be practiced. These are not necessarily congruent with the skills measured in formal studies on cognition. Moreover, some functions that are analyzed can be directly affected by continuous use and abuse of drugs, a practice that was present in the lives of some of these children.^40^^,^^41^

Considering the group in question, it cannot be concluded that the abnormal results from the behavioral auditory processing test reflected cognitive abnormalities. Even so, it is important that this hypothesis should be considered, given that some studies have indicated this problem. Moreover, it is also important that comparisons relating to the profile of the study group should be made such that populations of the studies conducted in other countries can also be taken into consideration. Therefore, it needs to be taken into account that there are cultural and economic differences between the studies cited here, which may in some manner interfere with the characteristic profile.

In addition to cognitive skills, the attention variable may also have influenced the results from the behavioral auditory processing tests. According to Alves,^1^ the street situation is also related to fluctuating attention levels consequent to the variety of stimuli present, exercising of work activities, care relating to too much exposure and constant mistrust.

Another important factor present in the study group was school complaints. It is known that this is the main reason for referring children for auditory processing evaluation,^18^ and this is in accordance with the great number of studies that have reported abnormalities relating to learning and auditory processing.^22^^,^^24^ The results from the present study therefore corroborate this hypothesis and show the importance of diagnosing and treating these abnormalities as a possible means of attempting to reduce school absenteeism.

## CONCLUSIONS

This study showed that children and adolescents in socially vulnerable situations had poor performance in comparison with a control group, in behavioral auditory processing tests, even though their auditory brainstem pathways were unimpaired, as shown by the normality of the BAEP results. The importance of this result lies mainly in repercussions from such abnormalities in relation to several characteristics of oral and written language acquisition and their interference in the quality of life of this population. Future research is required, in order to investigate better the manner in which the profile of this group can affect the abnormalities found.
